# The number of intestinal bacteria is not critical for the enhancement of antitumor activity and reduction of intestinal toxicity of irinotecan by the Chinese herbal medicine PHY906 (KD018)

**DOI:** 10.1186/1472-6882-14-490

**Published:** 2014-12-15

**Authors:** Wing Lam, Zaoli Jiang, Fulan Guan, Rong Hu, Shwu-Huey Liu, Edward Chu, Yung-Chi Cheng

**Affiliations:** Department of Pharmacology, Yale University School of Medicine, New Haven, CT 06510 USA; University of Pittsburgh Cancer Institute, University of Pittsburgh School of Medicine, Pittsburgh, PA 15232 USA

## Abstract

**Background:**

The four-herb Chinese medicine PHY906(KD018) has been shown to both enhance the *in vivo* antitumor activity of irinotecan (CPT-11) against colon cancer tumor allografts and alleviate intestinal toxicity caused by CPT-11.

**Methods:**

Since intestinal bacteria can metabolize CPT-11 and PHY906, we investigated whether intestinal bacteria play a critical role in the *in vivo* activity of PHY906 in murine Colon-38 tumor-bearing mice. Intestinal bacteria were depleted using streptomycin/neomycin for 10 days before and during treatment with PHY906 and/or CPT-11. qPCR using 16S DNA group-specific primers was used to quantify the levels of the major intestinal bacteria.

**Results:**

Both PHY906 and antibiotic treatment changed the profile of intestinal bacteria species: *Lactobacillus/Enterococcus, Bacteroides, Clostridium leptum, and E. rectale/C. coccoides.* Antibiotic treatment did not alter the ability of PHY906 to enhance the antitumor activity of CPT-11. Antibiotic treatment alone partially reduced animal body weight loss in CPT-11-treated mice. However, PHY906 treatment was able to protect against the body weight loss in the CPT-11/antibiotic treatment group. H&E and PCNA staining of intestine showed that antibiotic treatment partially reduced the intestinal damage caused by CPT-11 but not as effectively as PHY906 treatment. Antibiotic treatment plus PHY906 conferred the most effective protection of intestine histological structure against damage by CPT-11. Both PHY906 and antibiotic treatment inhibited CPT-11-associated inflammatory processes, including infiltration of the intestine by neutrophils, MCP1 and TNF-alpha mRNA expression in the intestine, and expression of pro-inflammatory cytokines G-CSF and MCP1 proteins in the plasma. However, whereas antibiotic treatment suppressed the mRNA expression of two important intestinal progenitor/stem cell markers, Olfm4 and Lgr5, PHY906 treatment resulted in enhanced expression of these two stem cell markers.

**Conclusions:**

Alterations in the population of intestinal bacteria did not affect the abilities of PHY906 to enhance CPT-11 antitumor activity or reduce the intestinal toxicity associated with CPT-11 treatment. The major species of intestinal bacteria do not appear to play a role in PHY906’s enhancement of the therapeutic index of CPT-11 in tumor-bearing mice. Thus, patients with different intestinal bacterial profiles may still benefit from PHY906 treatment alongside CPT-11.

## Background

PHY906 (KD018) is based on the formulation Huang Qin Tang (HQT), which consists of four main herbs: *Glycyrrhiza uralensis* Fisch (**G**), *Paeonia lactiflora* Pall (**P**), *Scutellaria baicalensis* Georgi (**S**), and *Ziziphus jujuba* Mill (**Z**). Huang Qin Tang has been used for over 1800 years to treat a variety of gastrointestinal symptoms including diarrhea, nausea and vomiting, and abdominal cramps. PHY906 is manufactured using high-quality herbs picked by experienced herbalists and manufactured following cGMP (current Good Manufacturing Practice). Consistent preparations of PHY906 have been made over a period of 10 years as documented by Phytomics QC using standardized chemical and biological fingerprints [1].

Irinotecan (CPT-11) in combination with 5-fluorouracil (5-FU) and leucovorin continues to be used as the first-line therapy for treatment of metastatic colorectal cancer (mCRC). CPT-11 is also approved as a second-line monotherapy for recurrent mCRC following treatment with an oxaliplatin-based regimen. The dose-limiting toxicities of CPT-11 include nausea and vomiting, abdominal pain/cramps, and diarrhea. CPT-11 administration has also been shown to cause gastrointestinal bleeding, a symptom often associated with colonic ulceration resulting from intestinal cell death and inflammation (3). Once CPT-11 is administrated, it is converted to the active metabolite SN-38 (7-Ethyl-10-hydroxy-camptothecin) by hepatic and intestinal carboxyesterases [2–4]. SN-38 damages DNA in intestinal cells as well as tumor cells, and can trigger acute life-threatening diarrhea in patients [5]. In the liver, SN-38 is metabolized by hepatic UDP–glucuronyltransferase into the inactive SN-38G metabolite, which is excreted into the jejunum via the bile duct [6, 7]. Intestinal bacterial β-glucuronidase can convert SN-38G back into the SN-38 metabolite, which can directly damage the intestine and promote intestinal inflammation. Several approaches have been used to reduce GI toxicity resulting from CPT-11-based chemotherapy, including modification of the schedule of administration [8, 9], intestinal alkalization [10, 11], treatment with anti-diarrheal therapies [12–16], genetic testing [17], ABCB1 transporter inhibitor treatment [18, 19], treatment with enzyme (β-glucuronidase, UGT1A1, carboxylesterase, COX-2) inducers or inhibitors [20–23], antibiotic treatment [24–26], treatment with adsorbing agents [27, 28], and treatment with other agents [29–32]. However, to date, none of these approaches has been found to be of significant clinical benefit.

Since Huang Qin Tang has been used for over 1800 years ago to treat diarrhea, PHY906 was chosen as a potential treatment strategy to reduce the GI side effects associated with CPT-11 treatment. In *in vivo* pre-clinical studies, PHY906 reduced the GI toxicities caused by CPT-11 while simultaneously enhancing CPT-11’s *in vivo* antitumor activity [33]. Each of the four main herbs comprising the PHY906 formula was required to maximize both its cytoprotective and antitumor effects. A phase 1/2 randomized double-blinded, placebo-controlled clinical trial was conducted to examine PHY906’s clinical potential as an adjuvant to CPT-11, 5-fluorouracil, and leucovorin (5FU/LV) for the treatment of chemotherapy-naive mCRC patients. PHY906 treatment resulted in a significant decrease in the incidence of nausea/vomiting and diarrhea, and of note, treatment with PHY906 was not associated with any toxicity on its own [34, 35]. The pharmacokinetics of CPT-11 and 5FU, respectively, was not affected by PHY906 treatment [34].

PHY906 is orally administrated, and the individual herbal components can be metabolized and transformed by gut flora. Individuals may carry different profiles of intestinal bacteria in their GI system depending on gender [36], diet [37], age [38], and co-morbid illnesses [38, 39]. Therefore, the impact of PHY906 may be different for individuals with different spectra of intestinal flora. To test this hypothesis, we investigated the potential interaction of PHY906 and CPT-11 on intestinal damage and antitumor activity in mice with or without depletion of intestinal bacteria. Our findings demonstrate that the ability of PHY906 to increase the therapeutic index of PHY906 is not affected by levels of major bacteria species in the intestine.

## Methods

Murine Colon-38 cells were transplanted subcutaneously into four- to six-week-old female BDF1 mice (Charles River Laboratories, Wilmington, MA). After 10 to 14 days, mice with tumor sizes of 150–300 mm^3^ were selected. Intestinal bacteria were depleted using streptomycin/neomycin (200 mg/kg, p.o. bid) for 10 days before and during the treatment of PHY906 and/or CPT-11. PHY906 was given orally (p.o.) for four days (twice per day; b.i.d., 500 mg/kg) and CPT-11 (360 mg/kg) was administered intraperitioneally (i.p.) on day 0. On day 0, PHY906 was given 30 minutes prior to CPT-11 administration. Mice (BDF1 bearing Colon-38 tumors) were terminated by cervical dislocation on day 4. Total DNA of the middle jejunum and the colon were purified using DNeasy Blood & Tissue Kit (Qiagen, Germantown, MD) according to manufacturer's instructions. qPCR using 16S DNA group-specific primers for *Eubacteria, Lactobacillus/Enterococcus* group (**Lact**), *Bacteroides* group (**Bact**), *Clostridium leptum* group (**Clept**) and *E. rectale/C. coccoides* group (**Erec**) was used to quantify the major intestinal bacteria in the middle jejunum and the colon [40]. Middle jejunum tissues were removed, fixed in formalin, embedded in paraffin, and sectioned into 10 μm pieces. Immunohistochemistry was used to detect protein expression in the middle jejunum tissues. Apoptosis was quantified by cleaved caspase 3 staining [33]. Cell proliferation was determined by proliferating cell nuclear antigen (PCNA) staining [33]. Quantitative RT real-time PCR was used to quantify Lgr5, Olfm4, Bmi, TNFα, and MCP1 mRNA expression in the middle jejunum [33]. Cytokine expression in the plasma was measured using the BD^TM^CBA (Cytometric Bead Array) (BD biosciences, San Jose, CA) according to manufacturer's instructions [33]. Data were analyzed by one-way or two-way ANOVA (GraphPad Prism 5, San Diego, CA), Student’s T-test (Microsoft Office Excel), and correlation analysis (GraphPad Prism 4). Difference were considered to be statistically significant when P < 0.05. All animal experiments were carried out in accordance with an approved Yale University Institutional Animal Care and Use Committee (IACUC) protocol. Murine Colon 38 cell lines were provided by Dr. Giuseppe Pizzorno, Ph.D., Pharm.D (Translational Science, Nevada Cancer Institute, USA).

## Results

### Treatment with PHY906, antibiotics, and/or CPT-11 alters the profile of major intestinal bacteria species

**Figure 1:**
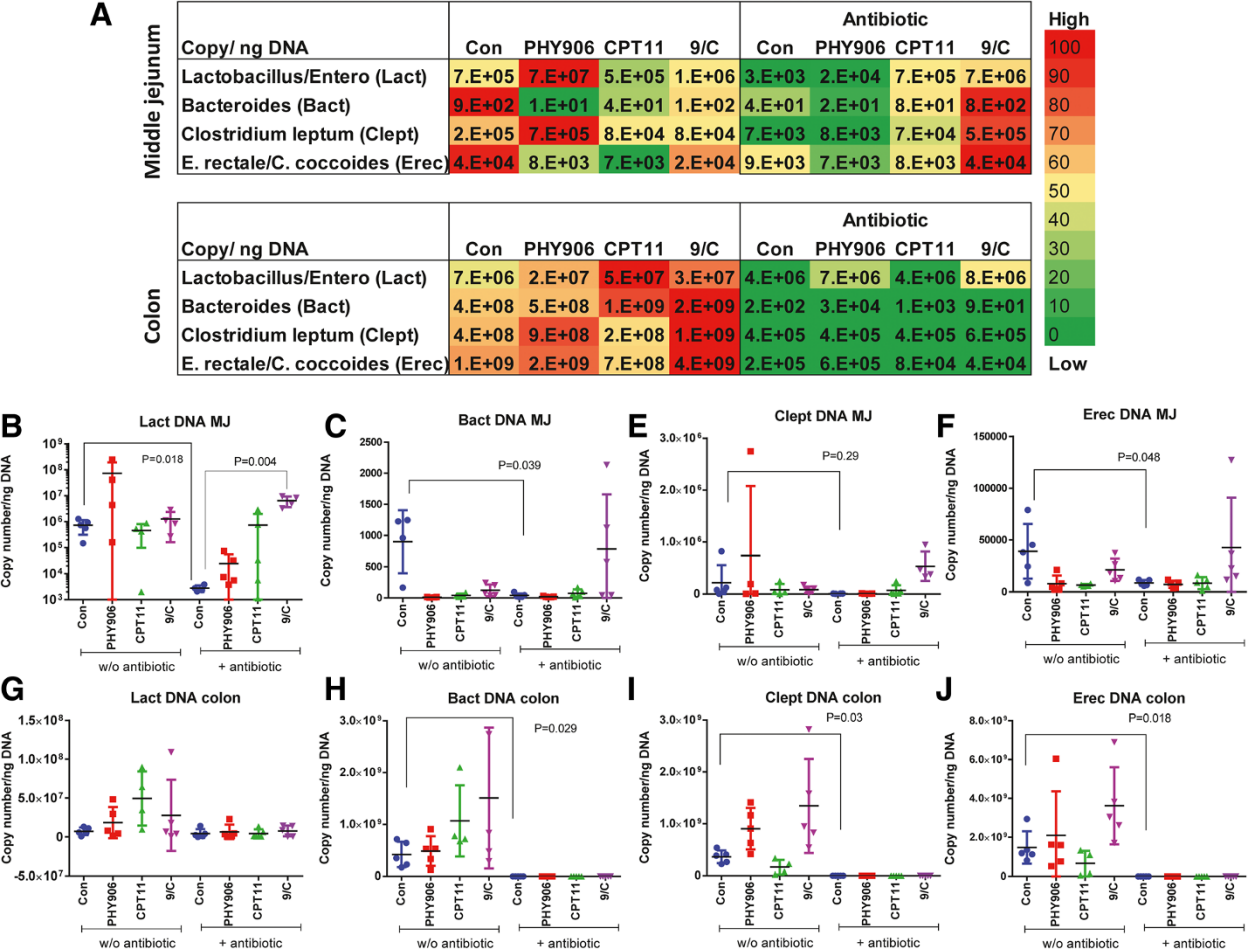
**Effect of antibiotics, PHY906, CPT-11 and PHY906/CPT-11(9/c) on intestinal bacteria densities in murine Colon-38 tumor-bearing BDF1 micex.** **(A)** Quantitative PCR heat maps for *Lactobacillus/Enterococcus* (Lact), *Bacteroides* (Bact), *Clostridium leptum* (Clept) and *E. rectale/C. coccoides* (Erec). The abundance of bacteria is categorized from high (red) to low (green) as indicated by the bar on the right side of the table. The top of the bar (100%) indicates the highest density of each bacterial group at each row of the table. **(B-J)** Quantitative PCR results for Lact, Bact, Clept and Erec in the middle jejunum (MJ) and colon. Each spot represents a mean of two or three different quantitative real-time PCR experiments (triplicate samples of each; 5 animals in each treatment group). Student's t-test was used to determine whether differences between treatment groups were significant. Details of experimental procedures are given in Methods.

Intestinal bacteria in Colon-38 tumor bearing BDF1 mice was depleted with streptomycin/neomycin (200 mg/kg, p.o. bid) for 10 days before (our preliminary result indicated 7-day pre-treatment was not sufficient to deplete intestinal bacteria, data not shown) treatment with PHY906 (500 mg, p.o. bid days 0–3) and/or CPT-11 (360 mg/kg, I.P. day 0). On day 4, qPCR was used to quantify the levels of major intestinal bacteria in the middle jejunum and in the colon, using 16S DNA group-specific primers for *Eubacteria, Lactobacillus/Enterococcus* group (**Lact**), *Bacteroides* group (**Bact**), *Clostridium leptum* group (**Clept**) and *E. rectale/C. coccoides* group (**Erec**). As seen in Figure 1, PHY906 treatment significantly decreased levels of **Bact** (P = 0.012) and **Erec** (P = 0.03) (Figure 1A, C, F) but caused a slight increase in **Lact** (Figure 1A, B). PHY906 treatment increased levels of **Clept** but not **Lact, Bact, or Erec** in the colon, and it had no effect on **Clept** levels in the middle jejunum (Figure 1A, I). CPT-11 treatment significantly reduced levels of **Bact** (P = 0.014) and **Erec** (P = 0.04) (Figure 1A, H, J) but not **Lact** or **Clept** in the middle jejunum. In the colon, CPT-11 increased the density of **Lact** (P = 0.03) (Figure 1A, G). Administration of PHY906 following CPT-11 treatment significantly increased levels of **Clept** (P = 0.04) and **Erec** (P = 0.03) (Figure 1A, I, J) but not **Lact** and **Bact** in the colon. In middle jejunum, PHY906 + CPT-11 treatment only increased the density of **Erec** (P = 0.03) (Figure 1A, F).

In the colon, antibiotic treatment depleted the density of **Bact**, **Clept**, and **Erec** by more than 99% but did not affect that of **Lact** (Figure 1A, G-J). In the middle jejunum, antibiotics reduced levels of **Lact**, **Bact**, and **Clept** by about 95% and decreased **Erec** by nearly 80% (Figure 1A-F). After antibiotic treatment, both PHY906 and CPT-11 increased the density of **Erec (**P = 0.02 and 0.04, respectively) (Figure 1A, J) but not **Lact, Bact,** or **Clept** in the colon; antibiotic + drug treatment also had no effect on the levels of tested bacteria in the middle jejunum. It is interesting to note that treatment with CPT-11, antibiotic, and PHY906 caused a significant increase in the density of **Lact** (P = 0.004) (Figure 1A, B) in the middle jejunum but not the colon. The level of **Lact** in CPT11/PHY906/antibiotic-treated mice was nearly 10-fold higher (P = 0.004) than that observed in the control group in antibiotic treatment conditions (Figure 1A, B).

Taken together, these studies show that PHY906 and CPT-11 with or without antibiotic treatment have variable effects on bacterial profiles in different regions of the intestine. Moreover, the interaction between PHY906 and CPT-11 could change bacterial profiles in the different segments of intestine.

### The effect of antibiotic treatment on the anti-tumor activities of CPT-11 and CPT-11/PHY906

**Figure 2:**
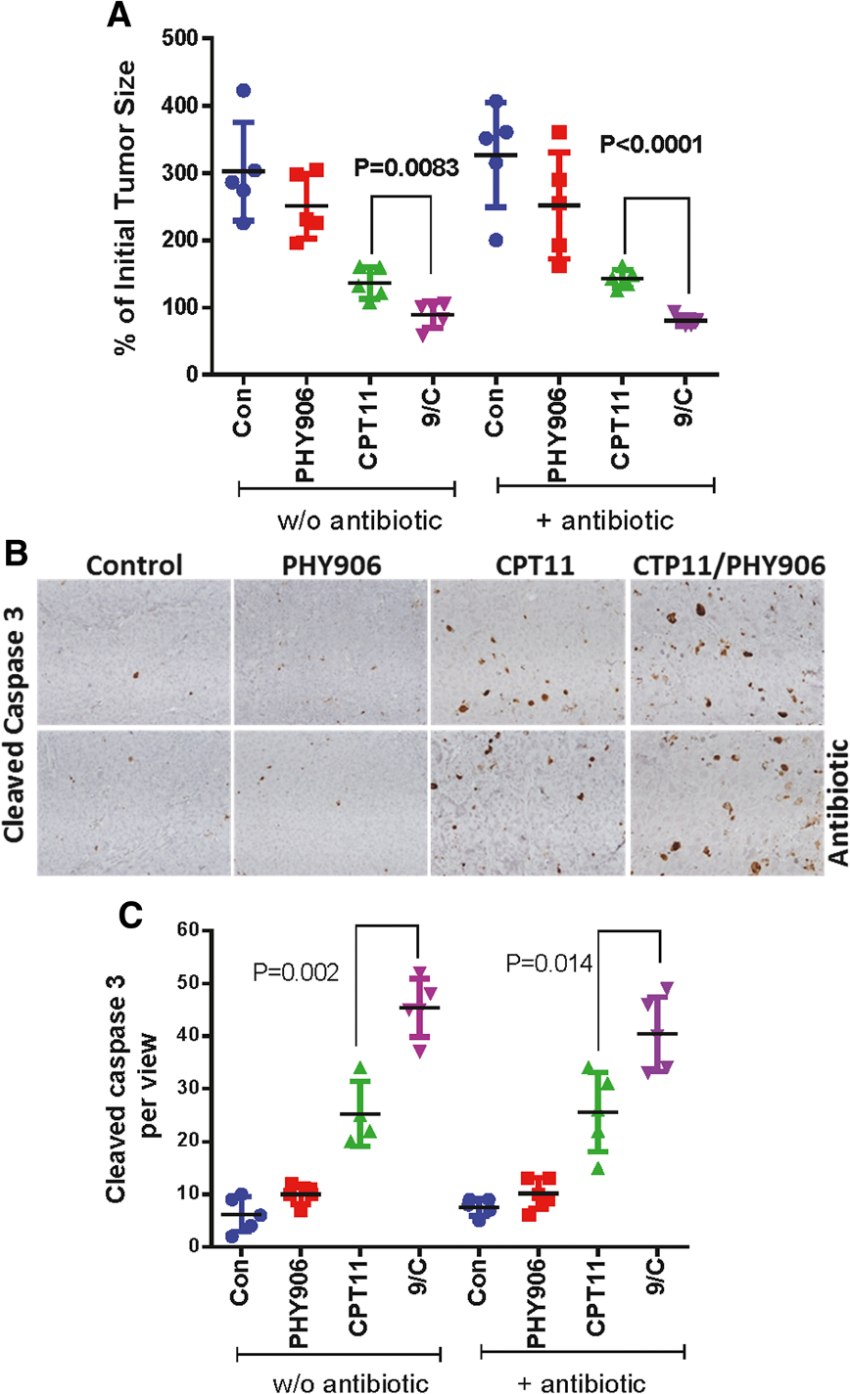
**Effect of antibiotic treatment on the anti-tumor activity and GI toxicity of PHY906, CPT-11 and PHY906/CPT-11(9/c) in murine Colon-38 tumor-bearing BDF1 mice. (A)** Effect of PHY906 and/or CPT-11 on the growth of Colon 38 tumor cells in BDF1 mice with or without (w/o) antibiotic treatment. Each spot represents an individual tumor’s size relative to that at initial treatment. One way ANOVA was used to determine the significance of the differences between curves **(B)** Immunohistochemistry staining for cleaved caspase-3 in Colon-38 tumor sections under different treatment conditions as indicated on the graph. Photographs in the figure were taken at 200× magnification. **(C)** Quantitation of cleaved caspase-3 staining. Cleaved caspase-3 stained cell per each view of Colon-38 tumor sections following different treatment conditions as indicated on the graph. Each spot represents the mean number of heavily stained cells from 4 to 5 views of each tumor section. Student's t-test was used to determine whether differences between treatment groups were significant. Details of the experimental procedures are given in Methods.

As it has been shown previously, treatment with PHY906 alone did not affect Colon-38 tumor growth. However, PHY906 was able to significantly enhance the antitumor activity of CPT-11 against Colon-38 cells (P < 0.0083) (Figure 2A). Treatment with antibiotics alone did not alter Colon-38 tumor growth (Figure 2A). Furthermore, antibiotic treatment did not affect the antitumor activity of either PHY906 or CPT-11 (Figure 2A). Most importantly, antibiotic treatment had no effect on the ability of PHY906 to significantly enhance the antitumor activity of CPT-11 against Colon-38 tumor growth (P < 0.0001). Cleaved caspase-3 staining indicated that PHY906 increased the level of apoptosis in Colon-38 tumors induced by CPT-11 with (P = 0.014) or without (P = 0.002) antibiotic treatment (Figure 2B, C).

### PHY906 reduced GI damage caused by CPT-11 regardless of antibiotic treatment

**Figure 3:**
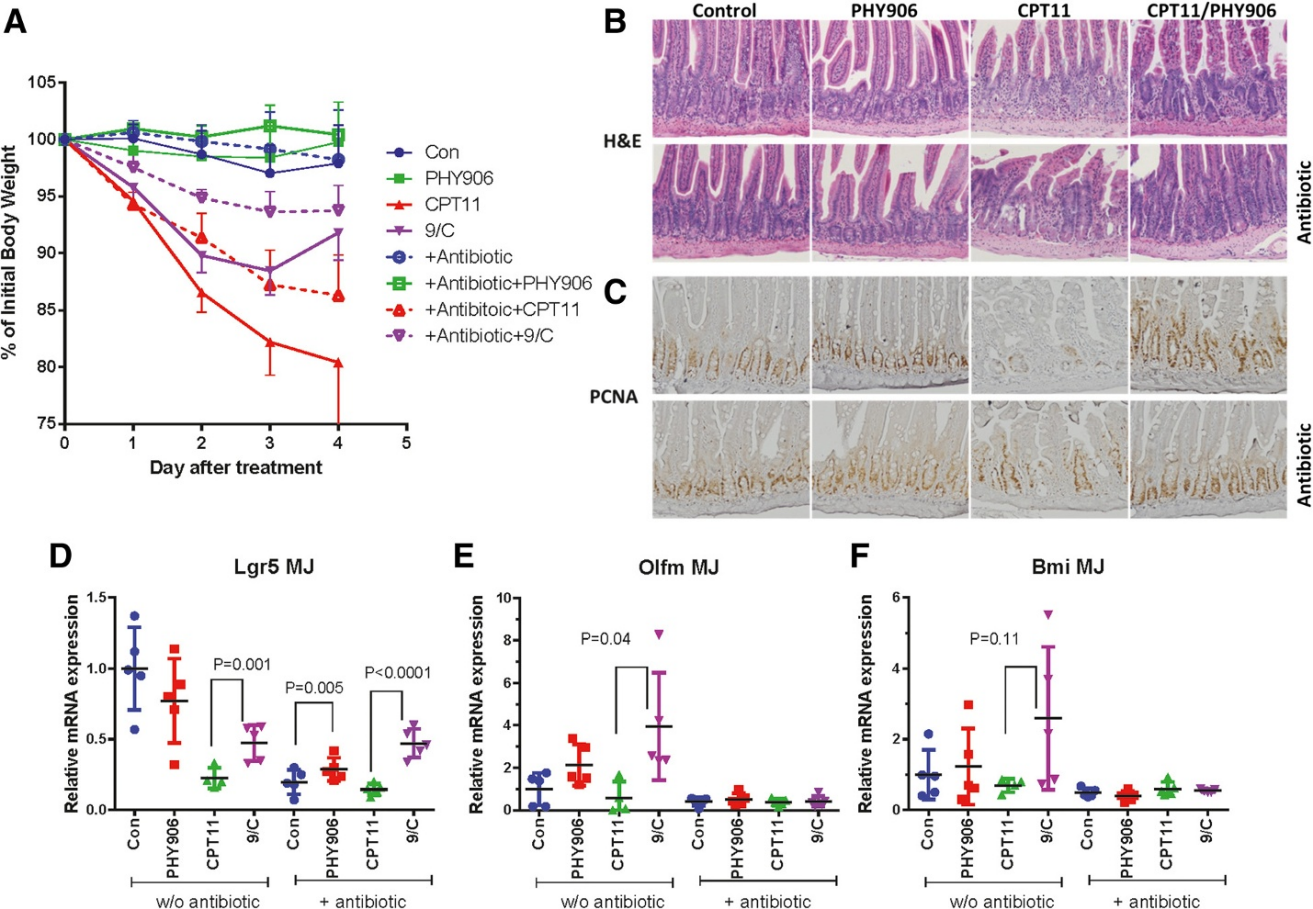
**Effect of PHY906 on CPT-11-induced body weight loss and damage of the middle jejunum in murine Colon-38 tumor bearing BDF1 mice with or without (w/o) antibiotic treatment on day 4. (A)** Effect of PHY906 in protecting against weight loss induced by CPT-11 with or without (w/o) antibiotic treatment. Error bars indicate standard deviations; N = 5. **(B)** Hematoxylin and eosin staining for visualizing the formalin fixed sections of the middle jejunum four days after treatments commenced. Photographs were taken at 200x magnification. **(C)** Effect of PHY906 on the expression of Proliferating Cell Nuclear Antigen (PCNA) following CPT-11 treatment with or without antibiotic treatment. **(D-F)** qPCR for the mRNA expression of the proposed intestinal stem cell markers Lgr5, Olfm4 and Bmi1 in the middle jejunum under different treatment conditions. β-actin was used as an internal control. Each spot represents the mean of two or three different experiments (triplicate samples of each; N = 5). Student's t-test was used to determine whether differences between treatment groups were significant. Details of experimental procedures are given in Methods.

PHY906 treatment did little to reverse the body weight loss caused by CPT-11 by day 2 but promoted a significant recovery in body weight by day 4 (Figure 3A), a result consistent with our previous findings [33]. Antibiotic treatment partially mitigated the body weight loss triggered by CPT-11 between day 2 and day 4 (P = 0.051) (Figure 3A). PHY906 further protected against CPT-11-triggered body weight loss regardless of antibiotic treatment (Figure 3A). There was no difference in animal body weight between the CPT-11/PHY906 group and the CPT-11/PHY906/antibiotic group on day 4 (Figure 3A); PHY906 alone could significantly protect against body weight loss following CPT-11 treatment on day 4 (Figure 3A). In conclusion, PHY906 was able to enhance the antitumor activity of CPT11 and protect against CPT11-triggered body weight loss in mice with different intestinal bacteria profiles.

Consistent with our previous report [33], mice treated with CPT-11/PHY906 displayed improved histology in the middle jejunum, especially in the crypt area, as compared to mice treated with CPT-11 alone (Figure 3B). PCNA staining also indicated that the majority of GI crypt cells in mice treated with CPT-11/PHY906 were actively proliferating (Figure 3C). Antibiotic treatment was able to protect some crypts from damage caused by CPT-11 but was not nearly as effective as PHY906 treatment alone (Figure 3B). PCNA staining showed only some crypt cells actively proliferating in mice treated with antibiotics plus CPT-11 (Figure 3C). PHY906 plus antibiotic treatment yielded the most pronounced improvement in the histology of the middle jejunum from damage caused by CPT-11 (Figure 3B). However, RT-qPCR results indicated that antibiotic treatment could affect two important intestinal progenitor/stem cell markers: Lgr5 and Olfm4 mRNA expression. Antibiotic treatment alone strongly inhibited Lgr5 mRNA expression (P = 0.001) to a level similar to that in CPT-11-treated mice (Figure 3D). However, antibiotic treatment did not affect PHY906’s induction of Lgr5 mRNA expression following CPT-11 treatment (P < 0.0001) (Figure 3D). Furthermore, although antibiotic treatment did not significantly inhibit Olfm4 or Bmi mRNA expression (Figure 3E, F), antibiotic treatment inhibited PHY906’s induction of Olfm4 mRNA expression following CPT-11 treatment (Figure 3E). These results suggest that antibiotic treatment or the profile of intestinal bacteria may in some manner affect the behavior of intestinal progenitor/stem cells.

### Anti-inflammation activity of PHY906 against CPT-11 toxicity is not affected by antibiotic treatment

**Figure 4:**
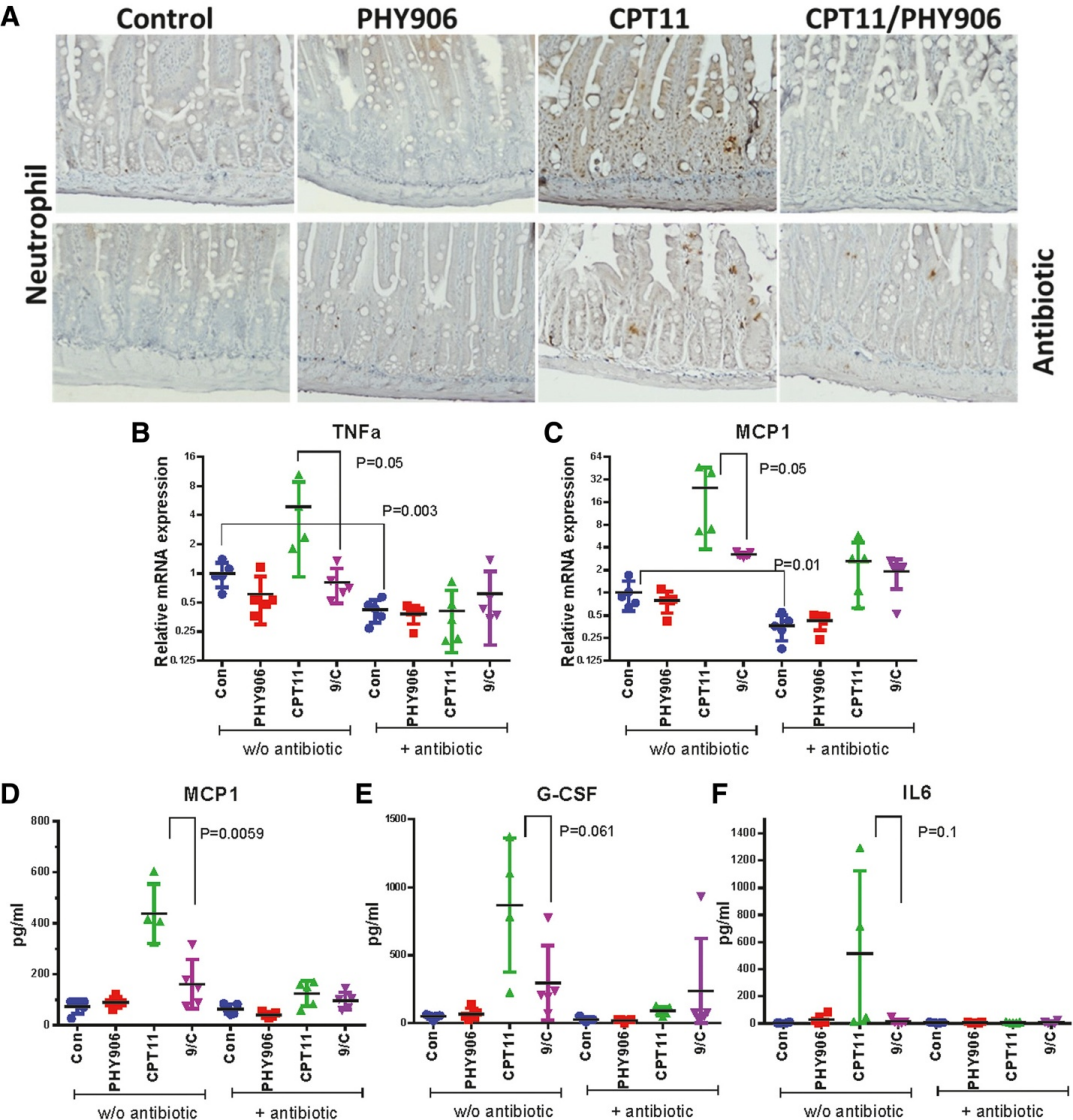
**Effect of PHY906 on CPT-11-induced inflammation in murine Colon-38 tumor-bearing BDF1 mice with or without (w/o) antibiotic treatment on day 4. (A)** Neutrophil infiltration in the middle jejunum section after different treatments. Photographs were taken at 200x magnification. **(B and C)** qPCR for TNFα and MCP-1 in the middle jejunum after treatment. β-actin was used as an internal control. Each spot represents the mean from two or three different experiments (triplicate samples of each; N = 5). **(D-F)** Detection of TNF-α, G-CSF and IL6 protein in the plasma after different treatments. (Each spot represents the mean from triplicate samples of each plasma sample; N = 5). Student's t-test was used to determine whether differences between treatment groups were significant. Details of experimental procedures are given in Methods.

As stated previously [33], PHY906 treatment inhibited several inflammatory processes triggered by CPT-11 in the middle jejunum, including neutrophil infiltration of the intestine (Figure 4A) and stimulation of TNF-α and MCP1 mRNA expression (Figure 4B and C). In the plasma, PHY906 reduced expression of pro-inflammatory cytokines induced by CPT-11 such as MCP1, G-CSF, and IL6 (Figure 4D-F). Antibiotic treatment also exhibited substantial anti-inflammatory activity in CPT-11-treated animals. It should be noted that 10-day pre-treatment with antibiotic significantly lowered the basal level of intestinal inflammation as reflected by a reduction in TNF-α (P = 0.003) and MCP1 (P = 0.01) mRNA (Figure 4B and C). However, antibiotic treatment did not affect basal levels of pro-inflammatory cytokines in the plasma.

## Discussion

The Chinese herbal medicine Huang-Qin Tang has a nearly 2000-year history of use for treatment of GI side effects such as diarrhea, nausea, vomiting, and abdominal cramps. However, the direct effect of Huang-Qin Tang on intestinal bacteria levels has never been formally investigated. Herein, we investigated the ability of PHY906, a standardized cGMP “Huang-Qin Tang” equivalent, to alter bacterial profiles differently in different segments of intestine. Our *in vivo* studies have shown that PHY906 does not act as a broad-spectrum antibiotic against the bacteria that were tested in our studies. For example, PHY906 treatment only inhibited **Bact** and **Erec** while antibiotic treatment decreased all kinds of tested bacteria in the middle jejunum. Thus, the underlying mechanism of Huang-Qin Tang for the treatment of diarrhea is completely different from that of anti-diarrheal antibiotics. However, one caveat is that PHY906 might still have antibiotic activity against certain bacteria that were not tested in the present study. Indeed, several studies have reported that individual herbs of PHY906 possess antibiotic activity. Different Chemicals of *Paeonia lactiflora* were found to have different activities against **Bact** [41] *.* It was found that human intestinal bacteria could transform flavonoids of *Scutellaria baicalensis* into aglycone-flavonoids, baicalein, oroxylin A, wogonin and norwogonin, each of which has different potencies against different types of intestinal bacteria [42]. Glycyrrhizol A and 6,8-diisoprenyl-5,7,4'-trihydroxyisoflavone (5) isolated form *Glycyrrhiza uralensis* were shown to exhibit potent antibacterial activity against *Streptococcus* mutants [43]. Crude ethanol extracts of *Ziziphus jujuba* fruits were also reported to exhibit antibacterial activity [44]. Future investigations, such as a comparison of the impacts of different herbal combinations of PHY906 on intestinal bacterial profiles, could help us to address which herb(s) or chemical(s) are responsible for which of PHY906’s antibiotic activities.

Previous studies have shown that treatment of PHY906 with *E. coli* β-glucuronidase alters the mixture’s bioactivities. PHY906 treated with β-glucuronidase was found to exhibit stronger Wnt3a potentiation activity and anti-TNFα activity but weaker anti-iNOS activity *in vitro* [33]. Thus, using antibiotics to deplete intestine bacteria, which have high β-glucuronidase activity, would be expected to have an antagonistic effect on PHY906’s biological activity. In addition, antibiotic treatment should also lead to reduced SN38 formation and damage of intestinal tissues. Streptomycin/neomycin treatment depleted over 90% of the major bacteria species in the gut. This finding is consistent with other reports in the literature [24, 45]. We observed that antibiotic treatment partially protected intestinal tissue from CPT-11 toxicity without enhancing CPT-11’s antitumor activity. In contrast, PHY906 was able to reduce the extent of GI damage while enhancing the antitumor activity of CPT-11. Thus, our studies suggest that PHY906 is able to maintain its biological activity in the presence of a wide range of intestinal bacteria and their respective β-glucuronidases. Perhaps human β-glucuronidase and UDP-glucuronosyltransferase (UGT), both of which are expressed in intestinal tissues, may impact PHY906 metabolism. In our phase I/ll clinical trial for treatment of mCRC patients, most plasma flavonoids from the orally administrated PHY906 were found to be glucuronidated, although some were sulfonated or methylated [46]. Our preliminary studies suggest that both human glucuronidase and UGT(s) play key roles in the metabolism of flavonoids in PHY906.

Treatment with either PHY906 or antibiotics may be able to reduce CPT-11-induced inflammation, including neutrophil infiltration of intestine, MCP1 and TNF-α mRNA expression in the intestine, and increased expression of pro-inflammatory cytokines G-CSF and MCP1 proteins in the plasma. However, it appears that PHY906 and antibiotics have different mechanism of action. It is known that intestinal bacteria are normally localized to the loose mucus and cannot penetrate the inner mucus layer [47–49]. However, damage to the mucus layer by CPT-11 allows intestinal bacteria to come in contact with epithelial cells or enter the blood stream, thus triggering the inflammatory process. Antibiotic treatment significantly reduces the level of intestinal bacteria and reduces the likelihood of bacteria-induced inflammation following CPT-11 treatment. This could also explain why antibiotic treatment alone reduced the basal level of MCP1 and TNF-α mRNA expression. In contrast, PHY906 accelerates the repopulation of epithelial cells, which restore the mucus layer and thus prevent bacterial penetration. PHY906 also suppresses inflammation by targeting several key signaling pathways, including NF-κB, iNOS, and COX2. For these reasons, the combination of antibiotics with PHY906 appears to more effectively suppress CPT-11-triggered inflammation and preserve the intestinal histological structure than either component alone. However, while antibiotic treatment did not affect PHY906’s induction of Lgr5 expression, it did suppress expression of intestinal progenitor/stem cell markers and inhibit PHY906’s ability to increase Olfm4 and Bmi levels following CPT-11 treatment. Lgr5 [50, 51], Olfm4 [52], and Bmi [53] all play important roles in maintaining the growth of intestinal progenitor/stem cells in the crypts of different segments of the intestine; intestinal bacteria can thus modulate intestinal stem cell proliferation to maintain gut homeostasis via the JAK–STAT and JNK pathways as demonstrated in *Drosophila* [54]. Therefore, the combination of PHY906 and antibiotics may not be the optimal approach to protect intestinal tissues from damage caused by CPT-11 or other toxic agents.

Inflammatory bowel diseases (IBD), including ulcerative colitis and Crohn’s disease, are caused by an innate immune response to luminal microflora in individuals with a certain genetic disposition. A recent finding indicated that the density of **Clept** is significantly reduced in the fecal microbiota of patients with Crohn’s disease and ulcerative colitis [39]. Our results showed that PHY906 alone can increase the density of **Clept** in colonic tissue. PHY906 is also able to maintain **Clept** and **Erec** levels in the colon following CPT-11 treatment. Therefore, PHY906 may have potential benefit in treating IBD by restoring the density of **Clept** in the colon. In addition, PHY906 inhibits NF-κB, COX2, and iNOS, all of which play key roles in IBD [55–57]. Thus, the potential use of PHY906 in treating IBD is worthy of further investigation.

## Conclusions

Our studies have shown that the depletion of intestinal bacteria by antibiotics does not impact the ability of PHY906 to enhance CPT-11’s antitumor activity and to protect against CPT-11-induced intestinal toxicity. Patients with a range of intestinal bacterial profiles may thus benefit from the use of PHY906 as a modulator of CPT-11-based treatment.

## Abbreviations

CPT-11: Irinotecan
qPCR: Quantitative real-time polymerase chain reaction
RT-qPCR: Reverse transcription quantitative real-time polymerase chain reaction
Lact: *Eubacteria, Lactobacillus/Enterococcus* group
Bact: *Bacteroides* group
Clept: *Clostridium leptum* group
Erec: *E. rectale/C. coccoides* group
mCRC: metastatic colorectal cancer
GI: Gastrointestinal
cGMP: current Good Manufacturing Practice
G: *Glycyrrhiza uralensis* Fisch
P: *Paeonia lactiflora* Pall
S: *Scutellaria baicalensis* Georgi
Z: *Ziziphus jujuba* Mill
MCP1: Monocyte Chemoattractant Protein-1
G-CSF: Granulocyte colony stimulating factor
iNOS: Inducible nitric oxide synthase
IBD: Inflammatory bowel disease.
